# Predictors of breath alcohol concentrations in college parties

**DOI:** 10.1186/s13011-017-0095-4

**Published:** 2017-03-30

**Authors:** Julie M. Croff, Eleanor Leavens, Kathleen Olson

**Affiliations:** 1 School of Applied Health and Educational Psychology, Oklahoma State University, 429 Willard Hall, Stillwater, OK 74078 USA; 2 Department of Psychology, Oklahoma State University, 116 North Murray, Stillwater, OK 74078 USA

**Keywords:** Intoxication, College, Student, Party, Consequence

## Abstract

**Background:**

Alcohol use and subsequent consequences are harmful for individual college students. Other students and the university can also be negatively impacted by the consequences of alcohol use.

**Method:**

A field-based study was used to assess the alcohol use environment at college parties. Researchers replicated a previous study by driving and walking a route to identify parties primarily on Thursday, Friday, and Saturday evenings between 9:00 PM and 1:00 AM across an academic year. Parties were randomly sampled. Hosts were asked for permission to enter the party at each sampled location. A census of partygoers was attempted at each party. Participants were asked to complete a brief survey and give a breath sample. All participants were recruited into a follow-up survey. Bivariate and multivariate analyses of individual-level and party-level factors associated with intoxication are presented.

**Results:**

The research team identified 29 parties: 16 were approached, and 12 were surveyed. Overall, 112 participants were surveyed for a response rate of approximately 28.7% of partygoers. Controlling for demographic characteristics, consumption of shots of liquor/spirits was significantly associated with a five times greater risk for intoxication. Notably, drinking games were protective of breath alcohol concentration (BrAC) risk in this model. Individuals who reported engaging in drinking games were 74% less likely to report a BrAC above the U.S. legal limit, while controlling for underage drinking in the model. Several party characteristics were identified that increased overall BrAC at the parties, including whether the party was themed, if it was a Greek life party, and whether there were illicit drugs present. Notably, when intoxication is examined by gender and party theme, women are significantly more likely to be intoxicated at themed parties: 75% were above 0.08 at themed parties compared to 35% above 0.08 at non-themed parties.

**Conclusions:**

Field-based data collection methods can, and should, be modified to conduct needs assessment and evaluation of prevention programs on college campuses. The findings on this campus were different than the originally sampled campus. Prevention programs should target unique risks identified on each campus, and to respond to problematic party behaviors with comprehensive programming rather than policy-level bans.

## Background

Negative consequences of alcohol consumption help drive university attrition. These consequences range from embarrassment and hangover to unwanted sexual activity, and in some cases even death [1]. In addition to the idiographic effects, alcohol-related consequences have the potential to disrupt the university community as a whole. Some researchers have even suggested that universities with higher rates of overall binge drinking also have higher rates of associated harm to non-binge drinking students [2, 3].

Attrition among undergraduate students is a particular concern because it is driven, at least in part, by underage alcohol use. Nearly three-quarters of college students are under the legal drinking age, and most (77.4%) have consumed alcohol in the past year [2]. Notably, prevalence of heavy episodic or binge use of alcohol in the previous two weeks is only slightly lower among underage students (over 43%) than students of legal drinking age (50%) [2].

College students are most likely to report consuming alcohol and engaging in heavy episodic drinking at off-campus parties and bars [2, 4]. Previous studies suggest that underage alcohol consumption is more likely to occur in environments conducive to alcohol use, including college parties. More than two-thirds of individuals surveyed at college parties were underage in one study [5]. While universities in the U.S. often prefer to use self-reported measures, like the National College Health Assessment (NCHA) and CORE drug and alcohol surveys, for benchmarking, the error introduced from paper-based or electronic self-report surveys with very low response rates may confound results. Given that off-campus parties are the most common location for underage college drinking, field research methodologies at college drinking parties may reduce errors associated with self-reported and retrospective research.

Field-based studies allow accurate measurement of risky behavior and consequences of this behavior; moreover, field studies allow identification of naturally occurring prevention strategies within unique college-drinking environments. Ecological theories suggest that environmental context of behavior is important for prevention programming: each campus population may adopt different naturally occurring prevention strategies. Continued measurement of the unique contexts of alcohol use across college campuses is critical to prevent consequences associated with excessive drinking in this population.

Individuals are poor reporters of past drinking behaviors, perhaps partially attributable to the cognitive and memory consequences of heavy episodic drinking [6–8]. Recall bias confounds calculation of estimated Blood Alcohol Concentration (eBAC); field-based studies have established this confound even when participants are asked to recall drinking within the context of the drinking event [5, 9]. Recalling the number of drinks consumed is only one part of error in calculation of eBAC. Measurement errors from retrospective survey research may also include difficulty in standardizing drink sizes, which may be more problematic when liquor and spirits are consumed. When naïve drinkers free pour spirits and consume alcohol in different shaped vessels they are likely to misjudge drink sizes [10, 11]. Since nearly half (44%) of heavy episodic drinking by underage drinkers includes spirits [12], errors in ability to respond to questions about standardized drink sizes can create substantial measurement error.

Collecting objective measures of breath alcohol concentration at underage college parties is a reliable and valid method for measuring alcohol use [5, 13, 14]. Studies on college drinking parties often emphasize a small subset of college students, such as parties hosted by Greek letter organizations [15, 16]. The largest study to date, by Clapp and colleagues [5, 9, 17], examined environmental cues for college drinking in natural, group settings, but was conducted at a single university. Results from this university may not generalize to other universities.

In an attempt to investigate whether predictors of were consistent across college drinking environments, this project aimed to replicate the college party sampling and survey method of Clapp and colleagues [6] at a large, public, rural university in the South Central United States. This study was replicated to identify whether alcohol-related risks at college parties are unique by university, or whether they might vary by region, or by universities with different prevention policies in place. Therefore, this study wanted to identify whether the same findings would occur from this research study. Specifically, we were interested in whether drinking game participation would increase BrAC at the individual level. Moreover, because students on this campus were more car dependent, we were eager to see if transportation to the event or transportation plans had an effect on drinking, and whether living on campus with protective policies had an effect on intoxication. At the party level, like prevous research, we were interested in whether themed events, loud parties, and late parties were significantly more likely to have intoxicated partygoers. Unlike previous research, the method presented in this manuscript has been modified to include a brief follow-up survey: the primary research aim of this study was to identify how high levels of measured intoxication impacted consequences, or whether harm reduction approaches were being naturally applied.

## Methods

### Identification & sampling of environments conducive to drinking

Objective measurement of college party environments was completed through random selection of parties. Parties, defined as five or more individuals at a social gathering, were systematically identified along a route surrounding campus. The route was established through the collaborative efforts of the research team, students, and local law enforcement. The 6-mile route included apartment complexes and residential neighborhoods with high rates of rental properties. Researchers drove and walked the route to identify parties primarily on Thursday, Friday, and Saturday evenings between 9:00 PM and 1:00 AM. These sampling days included one holiday evening associated with heavy drinking (Halloween).

Parties were identified by driving the complete route. It is essential for the study design that researchers drive the complete route in order to avoid oversampling at the beginning of the route. Upon identification and randomization, the study manager and one interviewer approached the party to recruit the host. If the party was no longer visible from the street or the party host denied the researchers entry, the next party on the randomized list was selected until an active party was identified. As with recruitment of individuals, refusal to participate may bias the sample.

Two to five researchers were able to accomplish data collection. Each night the research team consisted of at least one study manager and one interviewer. The study manager’s duties included party observation, safety awareness, interviewer management, and incentive distribution. On nights when more researchers were present, the additional researchers conducted interviews. To facilitate rapport and increase comfort levels of hosts, interviewer groups were ideally composed of both male and female research assistants and the majority of the party study team consisted of individuals in their early to mid-twenties.

Interviewers attempted a census of parties that had 20 or fewer partygoers. Interviewers recruited participants by briefly explaining the purpose of the study. Next, the interviewer explained the informed consent document. Once verbal consent was obtained, the interviewer requested that the participant stop drinking alcoholic beverages until a breath sample could be collected. This process allowed at least 10 min between their last drink and collection of the breath sample, a lag time necessary to greatly reduce or eliminate residual alcohol in the participant’s mouth, which would be likely to inflate measured breath alcohol content (BrAC).

### Party survey

The party survey included items regarding socio-demographic characteristics, transportation, motivations, alcohol consumption, and use and availability of illicit drugs. This brief survey consisted of 34-items. These survey items are a replication of the survey conducted by Clapp and colleagues [6]. The survey was completed through guided interview.

#### Socio-demographic characteristics

Socio-demographics were assessed by 7-items including gender, age, current student status, and race/ethnicity. If a participant reported being a student, follow-up questions included current class standing, greek-letter organization membership, and membership in an athletic team.

#### Transportation

Transportation to the party and planned transportation from the part were assessed by 2-items. Participants were asked how they got to the party that night, with options of I live here, drove myself, rode with someone else, walked, rode bike or skateboard, took public transportation, took a taxi or other. Participants were asked their plans for getting home that night with the same categories. This study took place before ride-share programs were common in the area.

#### Motivations

Motivations for attending the party were assessed by a single item, replicated from previous research [6], e.g. which of the following describes why you came here tonight? With response options of to socialize with friends, to meet a potential sexual partner, to have fun, to get drunk, to get in a fight, on a date, or other. Participants were encouraged to endorse all options that resonated with their motivations for being at the party.

#### Alcohol consumption

Current and planned alcohol consumption, how alcohol was obtained, and past two-week measures of heavy episodic drinking were also measured across 12-items. Participants were asked a yes/no question about whether they had consumed alcohol that night. If they endorsed this item, they were asked where they drank that night, where they got the alcohol they consumed, how many drinks they consumed in total and how many drinks at the party, how many drinks by alcohol type, and start and end time of drinking that day. Participants were also asked to report whether they planned to continue drinking, how intoxicated they felt, and how many occasions they had 4 (female)/5 (male) drinks in a row over the past two weeks.

#### Breath samples

Alcohol consumption was measured directly in the field using handheld breathalyzers (CMI Intoxilizer-400). Breathalyzers were calibrated once a month during the study period. Breath samples were completed by each participant at each party. When taken simultaneously, blood and breath samples are highly correlated (*r* = 0.95–0.98) [18]. Breath samples are considered to be as specific as blood samples: both are 100% specificity markers [19]. This commercial fuel-cell breathalyzer is available from the manufacturer, although pricing may preclude purchase by individuals.

#### Incentive

Each participant received a $5 gift card as an incentive.

### Novel methodological examination

While the original field method allowed for momentary ecological assessment, the method does not indicate whether BrAC, and therefore risks to the participant, increase or decrease after the survey. The original method fails to link the environmental context, the behavior, and consequences of the behavior. The addition of a follow-up survey allows the link from environment to experience with alcohol-related consequences. Measurement of consequences also allows evaluation of harm reduction strategies employed by partygoers.

During the field interview, immediately after consenting to participate, the interviewer recruited participants into a brief follow-up survey. If participants agreed to participate in the follow-up survey, they were asked to supply their phone number and first name. The form with this identifying information did not include any links to data collected in the field; it was immediately removed from the survey questionnaire and filed separately.

Follow-up surveys were conducted beginning the Monday after data collection and continued throughout the week. Follow-up survey items included measures of continued alcohol consumption (e.g., time of final drink, time to bed), subjective ratings of intoxication, transportation that night, smoking, and alcohol-related negative consequences, including risky sexual behavior.

During the first semester of data collection, research staff called participants three times per week (Monday, Wednesday, and Friday). Upon contacting participants, researchers conducted the follow-up survey by phone. Participants indicated their responses and research staff recorded them on an online survey database. Research participants seemed reluctant to discuss negative consequences associated with their alcohol use, like driving under the influence and engaging in unplanned sexual activity. The reluctance was evident in the initial completion rate of the follow-up survey of only 27.6% of participants. The protocol was revised in order to reduce attrition between the field and the follow-up survey. This revision included phone call reminders on Monday and Friday and sending the survey link via SMS text message on Monday, Wednesday, and Friday following data collection. This modification allowed participants the option to complete the survey without an interviewer and increased the completion rate of the follow-up survey to 58.3%.

### Analyses

College students who attend parties but refrain from consuming alcohol are important, but rare. In order to evaluate individual-level and party-level risk factors for consuming alcohol above the U.S. legal limit, analyses were restricted to participants who consumed alcohol and had a measurable BrAC at time of survey. Bivariate and multivariate logistic regression analyses on individual-level factors associated with risk for intoxication over the U.S. legal limit. Gender, race, drinking age, and Greek-letter organization status were included in the multivariate analysis in order to control for sociodemographic factors known to be related to alcohol consumption. Beyond these control variables, only covariates that were statistically significant in the bivariate analyses were included in the multivariate analysis. Frequencies of alcohol-related risks and consequences are presented from the follow-up data because the sample size is inadequate for bivariate analyses. Party-level factors were evaluated using bivariate techniques and pooled information from individual surveys.

## Results

On average, the team drove the complete route 2.63 (*SD* = 1.19) times each night. Completions driving the route ranged from one and five per night of data collection. The original method included only two route completions per night; in this study additional route completions were conducted in an attempt to identify at least one party. On a typical night when at least one party was identified, an average of 2.89 (*SD* = 1.27) parties were identified.

The research team identified 29 parties: 16 were approached, and 12 were surveyed. Seven parties were no longer in progress when the research team returned after completing the route. Law enforcement officials interrupted surveys at 4 of 12 (33.3%) parties after surveying began and arrived simultaneously with survey administration initiation at 1 of 16 (6%) parties approached. Surveys were discontinued upon arrival of law enforcement officials in each case. Therefore, 31.25% of parties identified could not be sampled because of law enforcement activity; this may have some influence on the 18.75% refusal rate because the research team was present at five parties where law enforcement was present, giving the team a negative association with law enforcement within the small party community.

Parties averaged 33.33 partygoers (SD = 25.88) based on research team counts for a total of approximately 400 partygoers across all 12 parties. Overall, 112 participants were surveyed for a response rate of approximately 28.7% of partygoers. The team did not keep track of the number of refusals by party. Typical reasons for refusal included being underage, suspicions due to the collection of breath samples, and not having time to participate.

The majority of participants were white (87.2%), male (67.9%) and under the legal drinking age (60.6%, see Table 1). Approximately two-thirds of participants were members of a fraternity or sorority. Nearly one in five participants reported driving themselves to the drinking event; very few, however, planned to drive home from the event. Most participants indicated motivations to drink until they were drunk. Breath samples indicated that the majority of participants over the U.S. legal limit of 0.08 (*n* = 60; 55.0%).

**Table 1:** Individual characteristics and bivariate characteristics associated with intoxication

Individual characteristics (level-1) (*n* = 109)					
Characteristics	% (n)	BrAC ≤ 0.079	BrAC ≥ 0.08	*χ* ^2^	*p*-value
		n (% within row)	n (% within row)		
Race					
White	87.2% (95)	43.2% (41)	56.8% (54)	0.96	0.33
Non-White	12.8% (14)	57.1% (8)	42.9% (6)		
Gender					
Female	32.1% (35)	16 (45.7%)	19 (54.3%)	0.12	0.91
Male	67.9% (74)	33 (44.6%)	41 (55.4%)		
Minimum Legal Drinking Age					
No	60.6% (66)	26 (39.4%)	40 (60.6%)	2.09	0.15
Yes	39.4% (43)	23 (53.5%)	20 (45.6%)		
Current college student					
No	6.4% (7)	1 (14.3%)	6 (85.7%)	2.84	0.092
Yes	93.6% (102)	48 (47.1%)	54 (52.9%)		
Fraternity or sorority member					
No	32.7% (33)	18 (54.5%)	15 (45.5%)	1.98	0.16
Yes	67.3% (68)	27 (39.7%)	41 (60.3%)		
Alcohol restrictions in housing					
No (off campus apt or house)	59.3% (64)	42.2% (27)	57.8% (37)	0.32	0.57
Yes (residence hall or Greek life)	40.7% (44)	47.7% (44)	52.3% (23)		
Played drinking game					
No	82.6% (90)	38 (42.4%)	52 (57.8%)	1.56	0.21
Yes	17.4% (18)	11 (57.9%)	8 (17.4%)		
Consumed shots					
No	63.6% (63)	34 (54.0%)	29 (46.0%)	11.25	0.001
Yes	36.4% (36)	7 (19.4%)	29 (80.6%)		
Motivated to get drunk					
No	81.7% (89)	45 (50.6%)	44 (49.4%)	6.16	0.013
Yes	18.3% (20)	4 (20.0%)	16 (80.0%)		
Planned mode of transportation to leave					
Safe Plan (stay, walk, etc.)	46.8% (51)	27 (52.9%)	24 (47.1%)	9.16	0.01
Ride with other	49.5% (54)	18 (33.3%)	36 (66.7%)		
Drive self	4 (3.7%)	4 (100%)	0 (0%)		
How much do you plan to drink? Enough to get…					
Not buzzed	8.3% (9)	9 (100%)	0 (0%)	19.52	0.001
Slight buzz	16.5% (18)	10 (55.6%)	8 (44.4%)		
Little drunk	27.5% (30)	16 (53.3%)	14 (46.7%)		
Very drunk	47.7% (52)	14 (26.9%)	38 (73.1%)		
How do you feel now					
Not buzzed	28.4% (31)	23 (74.2%)	8 (25.8%)	24.54	0.001
Slight buzz	42.4% (46)	22 (47.8%)	24 (52.2%)		
Little drunk	23.9% (26)	3 (11.5%)	23 (88.5%)		
Very drunk	5.5% (6)	1 (16.7%)	5 (83.3%)		

Bivariate analyses were conducted to identify specific factors contributing to BrAC above the U.S. legal limit (Table 1) . BrAC did not vary significantly by race (*χ* ^2 =^ 0.96, *p* = 0.33), gender (*χ* ^2^ = 0.12, *p* = 0.91), minimum legal drinking age (*χ* ^2^ = 2.09, *p* = 0.15), college student status (*χ* ^2^ = 2.84, *p* = .092), fraternity or sorority status (*χ* ^2^ = 1.98, *p* = 0.16), housing type based on alcohol policy (*χ* ^2^ = 0.32, *p* = .057), or drinking games (*χ* ^2^ = 1.56, *p* = 0.21). Drinking shots of liquor/spirits (*χ* ^2^ = 11.25, *p* ≤ 0.001), motivations to get drunk (*χ* ^2^ = 6.16, *p* = 0.013), a safe transportation plan (*χ* ^2^ = 9.16, *p* = 0.01), plans to become intoxicated (*χ* ^2^ = 19.52, *p* ≤ 0.001), and current intoxication level were significantly related to elevated BrAC (*χ* ^2^ = 24.54, *p* ≤ 0.001).

All variables were included in a logistic regression predicting elevated BrAC, excluding current intoxication report. Gender, race, age, and Greek-letter organization affiliation were retained in the reduced model to control for known population characteristics associated with heavy episodic drinking. The reduced model is presented in Table 2 and includes drinking games and consumption of 1.5 oz shots of liquor/spirits as predictive of intoxication. Consuming 1.5 oz shots of liquor/spirits was associated with approximately five times greater risk for intoxication. Notably, drinking games were protective of BrAC risk in this model. Individuals who reported engaging in drinking games were 74% less likely to report a BrAC above the U.S. legal limit, while controlling for underage drinking in the model.

**Table 2:** Reduced individual-level regression model of alcohol consuming partygoers predicting intoxication at or above the legal limit

Characteristic	OR (95% CI)	*p*-value
Gender		
Male	Ref.	0.391
Female	1.60 (0.55 – 4.63)	
Race		
White	Ref.	0.86
Non-white	0.88 (0.22 – 3.54)	
MLDA		
Underage		0.59
Legal Age	0.77 (0.29 - 2.01)	
Greek Life		
No	Ref.	0.25
Yes	0.53 (0.18 – 1.55)	
Shots		
No	Ref.	0.005
Yes	4.94 (1.61 – 15.10)	
Drinking Games		
No	Ref.	0.035
Yes	0.26 (0.075 – 0.91)

**Table 3:** Party characteristics

Party-level Characteristics (level-2) (*n* = 12)			
Characteristics	Average BrAC	t-value	*p*-value
Themed			
Yes	0.12	1.79	0.11
No	0.08		
Loud			
Yes	0.099	0.24	0.81
No	0.09		
Food Available			
Yes	0.08	0.37	0.72
No	0.097		
Drugs Present			
Yes	0.12	1.8	0.11
No	0.08		
Greek Life Party			
Yes	0.13	2.4	0.045
No	0.07		
Time of survey			
9:00 – 11:00 pm	0.077	1.93	0.088
11:00 pm – 2:00 am	0.13		

At follow up, all participants who endorsed drinking at the party also endorsed continued drinking after completing the baseline survey. Of the 21 participants retained at follow-up, none reported driving after drinking. Due to the small sample size, only descriptive statistics will be reported. A large proportion reported experiencing other alcohol-related negative consequences that night or the following day: becoming sick due to drinking (*n* = 2; 9.5%), passing out from drinking (*n* = 1; 4.8%), missing class/work (*n* = 1; 4.8%), experiencing a hangover (*n* = 8; 38.1%), drinking before breakfast the following morning (*n* = 1; 4.8%), and forgetting parts of the previous night (*n* = 4; 19.0%). Compared to participants under the legal drinking age, participants over the legal drinking age experienced more alcohol-related negative consequences. Additionally, individuals over the U.S. legal limit experienced more alcohol-related negative consequences compared to those under the U.S. legal limit.

Although sample sizes are too small for multi-level modeling, the party-level was evaluated independently, predicting mean BrAC at the party (Table 3). Several party characteristics were identified that increased overall BrAC at the parties, including whether the party was themed, if it was a Greek life party, and whether there were illicit drugs present. Of heavy drinking Greek Life parties, 3 of 5 were themed with illicit drugs present. Notably, when intoxication is examined by gender and party theme, women are significantly more likely to be intoxicated at themed parties: 75% were above 0.08 at themed parties compared to 35% above 0.08 at non-themed parties.

## Discussion

This study demonstrates application of a methodology that may be appropriate for needs assessment and evaluation of alcohol prevention programs near college campuses. The drinking environment, including the policy environment, has been shown to directly influence alcohol use by college students. As such, differences in the environment across college campuses may support differing programmatic needs.

### Individual-level

This study did not identify underage use as a significant predictor of BrAC, like other studies [see [20]]. Nor did this study identify common gender differences in intoxication by gender [21]. However, this study indicates that consumption of 1.5 oz shots of liquor/spirits is associated with a significant increase in the likelihood of being over the legal drinking limit. Harm reduction approaches or protective behavioral strategies may include watering drinks down with ice, alternating with mock-cocktails, recipes for cocktails made with beer, or planning for a safe ride home. Programing recommendations should address motivations to get drunk, as these are likely to influence consumption of 1.5 oz shots of liquor/spirits. Approaches which follow individuals through consequences and focus on behavioral change in the future may be most effective. For example, this method could be modified to give feedback during the follow-up phone call in order to change behavior at a time point when individuals may be motivated to change future behaviors [22–24].

Moreover, this study identified that drinking games were associated with reductions in intoxication. This may have occurred because drinking games common to this population include consumption of beer or because the drinking games being played include alcohol consumption over a longer time period. Widely available beer for underage drinkers in this sample is 4% alcohol by volume, therefore, consumption of small amounts of this beer may be protective of intoxication. As beer-based drinking games gain popularity, studies may continue to find that 1.5 oz shots of liquor/spirits are the real predictor of risk, not drinking games.

### Party-level

Risk at the party level may be most meaningful for informing prevention programs. This study was conducted at a university that does not permit alcohol in Greek-letter organization houses. This policy is meant to dissuade Greek-letter organization parties. However, the results of the current study indicate that Greek-letter organization parties are occurring outside of campus houses and that these parties have among the highest levels of measured intoxication, and may be particularly risky for females. The time of the party was also significantly related to measured intoxication; but, this is to be expected since partygoers have had longer period of time to consume alcohol.

Risks associated with themed events, particularly among young women have been documented in previous studies [9]. This study, combined with previous research, indicate that identification of themed events may be ripe for prevention programs. Notably, all three studies support need for targeted prevention for college women drinking at themed events.

### Consequences

As noted by White & Hingson, college students may be poor reporters of alcohol use, but they are able to more accurately self-report consequences associated with alcohol use [25]. Hangovers were the most commonly reported alcohol related consequence in the follow-up survey. Due to a relatively low follow-up rate, we were underpowered for some analyses regarding consequences. Future research should test and employ the use of text messaging to complete research necessitating follow-up data among college student populations. This may be particularly salient with alcohol using college students. Monitoring of consequences of alcohol use may be particularly helpful in creating behavior change programs.

### Method

This methodology may be appropriate for universities to implement for needs assessment and program evaluation. Modifications may be necessary for rural communities, colder climates or college towns. Measures of alcohol use and party environments from various campuses will increase ability to identify natural prevention strategies and environmental risks. Future field research methods must continue to include objective measurement of alcohol use. Previous research supports the relationship between party environments (e.g., party size, presence of food, rowdy behavior) and errors in estimated BACs among party guests [26], further amplifying the need for objective, momentary measurement of alcohol consumption at parties. Advances in technology may allow further reductions to staffing needed to accomplish this work, including the use of ecological momentary assessment coupled with transdermal alcohol sensors with GPS monitoring. These methodological changes, however, must be paired with the use of cell phone photos of the party environment in order to adequately link party environment to drinking behavior and alcohol-related negative consequences.

Several important lessons from this study may be helpful in the translation of this research method to practice. First, this data collection can be accomplished with small teams of graduate and undergraduate researchers. This project was completed with far fewer staff than the original study conducted by Clapp and colleagues [5, 6].

Second, seasonality may be a concern for conduction of this type of work. The research team did experience some problems associated with identifying parties. Clapp and colleagues [5, 6] conducted their field party research in a temperate climate, which may facilitate party identification due to outdoor parties, or through open windows and doors. Temperature control (heating and air conditioning) is common in homes through all seasons in South Central states, as such, it is more difficult to identify parties occurring inside homes with double pane windows and closed doors. Moreover, in neighborhoods where many students reside and there are multiple cars parked in front, identifiers for parties in progress are limited. Data collection was suspended during the colder winter months due to an inability for the research team to identify parties or conduct surveys outdoors.

Third, higher education and prevention professionals hoping to implement party programming may wish to contact local law enforcement officials and work in partnership so that the two activities do not conflict. During this study period, the research team worked independently from local law enforcement; therefore, 31.25% of parties identified could not be sampled because of law enforcement activity. Interruptions by law enforcement officials may be a major barrier to future field research as students may associate the survey or survey team with police presence and become fearful or distrusting of the researchers. Law enforcement activity coinciding with the project may have some influence on the 18.75% refusal rate at the party host level.

Finally, the addition of the follow-up point of contact provides an opportunity for prevention programming. The research team was most successful reaching students via text message to complete the follow-up survey, as participants were reluctant and perhaps self-conscious about reporting problems associated with alcohol use via telephone interview. Reports of negative consequences by students may allow referral to prevention programming. The inclusion of a follow-up survey with links to prevention resources is a worthwhile way to increase at-risk population participation in prevention activities. Allowing participants to complete the survey online or on their cell phone substantially increased follow-up rates and will be instrumental in assessing longer-term negative outcomes, harm-reduction strategies, and linking students to prevention resources in future programs.

### Limitations

Identification of parties and attrition presented problems for the research team. Party identification was made difficult due to weather, police presence, and the mobility of partygoers. In order to overcome these problems, future research may benefit from recruiting party hosts prior to parties. In spite of this, the current research was fully powered at baseline. Follow-up rates were relatively low in the current study; however, recent research highlights an overall decline in follow-up response rates among college students [27, 28]. This refusal rate could create a sampling bias. To improve the design, subsequent research could provide electronic questionnaires via text message and email, rather than phone surveys. Moreover, researchers could ensure monetary incentives are sufficient enough to elicit interested and participation in this important piece of the research project.

The sampling frame for this project includes recruiting parties first and individuals within parties second. Therefore, all individuals recruited are nested within clusters of parties. The results of this study indicate that there are differences in BrAC by party type, particularly when comparing Greek-letter organization parties to other parties. The sample size in this study precluded use of hierarchical modeling methods. Therefore, a limitation of this research is the nested nature of the observations at the individual level.

Finally, participants may have been sampled more than once at different parties. The team collected data across an academic year and may have had opportunities to sample the same individual (s) at multiple drinking events. The unique ID method used by this study included birth month and day, last four digits of phone number, and number of siblings. Based on this information there were not duplications in sampling over the study period, but, it is possible that the participant did not answer truthfully.

## Conclusion

In conclusion, field-based methods may be helpful for assessing the alcohol-consumption environment of each university. The context of policies for preventing alcohol-related problems and the norms around alcohol use may change the risk environment from place to place or at one time over another. This study was unique in its identification of drinking games as protective, which, demonstrates how the state-level alcohol policies for low-point (3.2% alcohol by weight) may create synergies with beer-based drinking games. Moreover, this research suggests that alcohol bans are likely to be unsuccessful among college students; prevention programs sometimes benefit from having a known location for risk environments, like Greek-letter organization housing. Regardless of use of breath test devices, follow-up regarding consequences of alcohol use can be helpful for creation of individualized prevention programs.

## References

[1] Hingson RW, Zha W, Weitzman ER (2009). Magnitude of and trends in alcohol-related mortality and morbidity among U.S. college students ages 18–24, 1998–2005. J Stud Alcohol Drugs Suppl.

[2] Wechsler H, Lee JE, Kuo M, Seibring M, Nelson TF, Lee H (2002). Trends in college binge drinking during a period of increased prevention efforts: findings from 4 harvard school of public health college alcohol study surveys: 1993–2001. J Am Coll Health.

[3] Wechsler H, Davenport A, Dowdall G, Moeykens B, Castillo S (1994). Health and behavioral consequences of binge drinking in college: a national survey of students at 140 campuses. JAMA.

[4] Wechsler H, Nelson TF (2008). What we have learned from the harvard school of public health college alcohol study: focusing attention on college student alcohol consumption and the environmental conditions that promote it. J Stud Alcohol Drugs.

[5] Clapp JD, Min JW, Shillington AM, Reed MB, Ketchie Croff JM (2008). Person and environment predictors of blood alcohol concentrations: a multi-level study of college parties. Alcohol Clin Exp Res.

[6] Clapp JD, Holmes MR, Reed MB, Shillington AM, Freisthler B, Lange JE (2007). Measuring college students’ alcohol consumption in natural drinking environments field methodologies for bars and parties. Eval Rev.

[7] Clapp JD, Reed MB, Min JW, Shillington AM, Croff JM, Holmes MR, Trim RS (2009). Blood alcohol concentrations among bar patrons: a multi-level study of drinking behavior. Drug Alcohol Depend.

[8] Weissenborn R, Duka T (2003). Acute alcohol effects on cognitive function in social drinkers: their relationship to drinking habits. Psychopharmacology (Berl).

[9] Clapp JD, Ketchie JM, Reed MB, Shillington AM, Lange JE, Holmes MR (2008). Three exploratory studies of college theme parties. Drug Alcohol Rev.

[10] Attwood AS, Scott-Samuel NE, Stothart G, Munafò MR. Glass shape influences consumption rate for alcoholic beverages. PLoS One. 2012;7(8):e43007–7. doi:10.1371/journal.pone.0043007. 10.1371/journal.pone.0043007 PMC3422221 22912776

[11] Del Boca FK, Darkes J (2003). The validity of self-reports of alcohol consumption: State of the science and challenges for research. Addiction.

[12] Naimi TS, Siegel M, DeJong W, O’Doherty C, Jernigan D (2015). Beverage-and brand-specific binge alcohol consumption among underage youth in the US. J Subst Use.

[13] Kypri K, Langley J, Stephenson S (2005). Episode-centered analysis of drinking to intoxication in university students. Alcohol Alcohol.

[14] Thombs DL, O'Mara RJ, Tsukamoto M, Rossheim ME, Weiler RM, Merves ML, Goldberger BA (2010). Event-level analyses of energy drink consumption and alcohol intoxication in bar patrons. Addict Behav.

[15] Geller ES, Kalsher MJ (1990). Environmental determinants of party drinking: bartenders vs. Self-service. Environ Behav.

[16] Geller ES, Clarke SW, Kalsher MJ (1991). Knowing when to say when: a simple assessment of alcohol impairment. J Behav Anal.

[17] Clapp JD, Min JW, Shillington AM, Reed MB, Lange JE, Holmes MR (2006). Environmental and individual predictors of error in field estimates of blood alcohol concentration: a multilevel analysis. J Stud Alcohol.

[18] Jones AW (2000). Medicolegal alcohol determination-blood-or breath-alcohol concentration?. Forensic Sci Rev.

[19] Marques PR, Voas RB (2005). Interlock BAC tests, alcohol biomarkers, and motivational interviewing: methods for detecting and changing high-risk offenders. Alcohol Ignition Interlock Devices.

[20] Montauti SB, Bulmer SM (2014). A research update on correlates of heavy episodic drinking among undergraduate college students. Am J Health Ed.

[21] Mahalik JR, Lombardi CM, Sims J, Coley RL, Lynch AD (2015). Gender, male-typicality, and social norms predicting adolescent alcohol intoxication and marijuana use. Soc Sci Med.

[22] Walters ST, Neighbors C (2005). Feedback interventions for college alcohol misuse: what, why and for whom?. Addict Behav.

[23] Carey KB, Scott-Sheldon LA, Carey MP, DeMartini KS (2007). Individual-level interventions to reduce college student drinking: a meta-analytic review. Addict Behav.

[24] Baer JS, Kivlahan DR, Blume AW, McKnight P, Marlatt GA (2001). Brief intervention for heavy-drinking college students: 4-year follow-up and natural history. Am J Public Health.

[25] White A, Hingson R (2014). The burden of alcohol use: excessive alcohol consumption and related consequences among college students. Alcohol Res.

[26] Clapp JD, Reed MB, Holmes MR, Lange JE, Voas RB (2006). Drunk in public, drunk in private: the relationship between college students, drinking environments and alcohol consumption. Am J Drug Alcohol Abuse.

[27] Baruch Y (1999). Response rate in academic studies-a comparative analysis. Hum Relat.

[28] Dey EL (1997). Working with low survey response rates: the efficacy of weighting adjustments. Res High Educ.

